# Awareness of Salt Intake among Community-Dwelling Elderly at Coastal Area: The Role of Public Health Access Program

**DOI:** 10.1155/2020/8793869

**Published:** 2020-02-21

**Authors:** Farapti Farapti, Aprilia Devi Fatimah, Erni Astutik, Atik Choirul Hidajah, Thinni Nurul Rochmah

**Affiliations:** ^1^Department of Nutrition, Faculty of Public Health, Universitas Airlangga, Surabaya 60115, Indonesia; ^2^Department of Epidemiology, Faculty of Public Health, Universitas Airlangga, Surabaya 60115, Indonesia; ^3^Department of Health Policy and Administration, Faculty of Public Health, Universitas Airlangga, Surabaya 60115, Indonesia

## Abstract

A geographical location such as coastal area is known as risk factor hypertension relating to high exposure of salty foods. Public health access had significant effect on reducing salt intake at the community level. This study assesses salt intake in older women resident at urban coastal in Indonesia participating in the public health program. This was a cross-sectional study involving older women (56.98 ± 5.7 years) resident at urban coastal in Kenjeran, Surabaya, Indonesia. Salt intake was calculated and estimated based on 24-h urinary sodium. The mean daily salt intake was 6.16 ± 3.48 g/d; only 11.8% of subjects consumed salt intake <3 g/day. However, majority of subjects (62.8%) consume salt <6 g/d. Awareness and participation were associated significantly with low salt intake. A significant association between participation, awareness, and salt intake may suggest that participating regularly in the public health program might cause our subjects controlled excessive salt intake by limiting their salt consumption. Since daily salt intake is still significant high and hypertension is still prevalence, comprehensive strategies to reduce salt should be considered in development of sodium-reduction initiatives in this region.

## 1. Introduction

Hypertension is a major public health problem worldwide because the prevalence continues to rise. The rising incidence of HT contributes to cause morbidity and mortality since hypertension is well recognized and the most common risk factors for cardiovascular and cerebrovascular disease [1]. The prevalence of hypertension is predicted to rise around 60% by the year of 2025 with nearly 1.5 million deaths (9.4% of total deaths) annually [2, 3]. Indonesian National Health Survey 2018 demonstrated that 34.11% of the Indonesian adults have established hypertension, and the prevalence continues to rise with increasing age [4].

Risk factors of HT are closely related to modifiable factors such as dietary habits particularly excess dietary salt. Most population studies provided evidence that daily salt intake in most countries is well above the World Health Organization (WHO) recommendation. Moreover, accumulating evidence strongly supports a detrimental effect of excess salt intake on blood pressure [5]. Decreasing the burden of diseases associated with hypertension has been identified as a public health priority, and the growing burden of hypertension increased awareness to reduce dietary salt [2, 6].

Salt-reduction strategy programs have been shown to be low cost and effective [6]. Several countries have already successfully established to reduce dietary salt in their populations including Japan, Finland, and more the United Kingdom [7]. Public health care, the leading health sector in the community, has an important role to promote population-level salt reduction by raising consumer awareness regarding salt intake, and the access to healthcare has an important role to public health outcomes [8]. Some evidence showed how public health access had a significant effect on health improvement including reducing salt intake at the population level [9]. Salt-reduction programs should seek to target not only in the common population but also especially in the most vulnerable groups [10].

Urban coastal dwellers are categorized as a vulnerable group since a geographical location such as coastal area is known as risk factors of hypertension relating to the high consumption of salty foods. The high reserves in salted fish consumed by the community can have a harmful effect on blood pressure [11]. The difficulty in accurately measuring salt intake using dietary assessment and since the majority of sodium/salt excreted in the urine, a 24-h urine collection is considered as the “gold standard” method to assess salt intake in the population; however, it is high in cost and less feasible [12]. Since our subjects were categorized as vulnerable population related to high salt intake and public health access has an important role to the health outcome, our study aims to investigate salt intake in older women resident at urban coastal in Indonesia participating in the public health program and analyze the factors related to it.

## 2. Materials and Methods

### 2.1. Design and Study Measurements

This was an observational study with cross-sectional design involving community-dwelling elderly at coastal area in 2015-2016. Data collection process was done by interviewing a structure questionnaire, measuring blood pressure, performing anthropometric data, and collecting a 24-h single urine sample. A structured questionnaire of demographic information and medical history were obtained from medical records; meanwhile, the consumption of hypertension drug use, awareness, participation, and salt preference to support the role of the public health program were directly fulfilled by participants.

### 2.2. Participants

We recruited subjects from the urban coastal community living in Kenjeran beach, Surabaya, aged ≥45 years old who met the study criteria. Fifty-one subjects who met the study criteria were obtained by consecutive sampling. Participants were included in the study if they were a member of elderly primary care setting, postmenopause women, dweller in coastal area for more than 10 years, and agree to collect a 24-h urine sample. We recruited only women because most of the men living in the coastal area (study location) worked as fishermen, so it was difficult for them to collect the 24-h sample accurately. In addition, only women were recruited because most of members (88%) of elderly health service are female. Participants with kidney dysfunction (creatinine clearance test (CCT) < 60 mL/min), cognitive impairment which was proven by mini-mental state examination (MMSE) score <24, consuming tobacco and alcohol, and inaccurate urine collection were excluded. Ethical approval was obtained from the Ethical Committee of Faculty of Public Health, Universitas Airlangga, and written informed consent was obtained from all subjects.

### 2.3. Public Health Program

To examine the role of the elderly public health program on salt intake, we only recruited subjects living near around the public health center and joining at the elderly public health program facilitated by the Surabaya government and for minimal 3 years. The health center is located in metropolitan city, Surabaya, which is the second biggest city in Indonesia. This program involves interprofessional health physicians such as nurses, general practitioners, nutritionists, and health counselors to provide care for older women in the region. The program was held 2-3 times per month. The variables of the public health program related to salt intake include awareness and participation, as well as the salt preference.  Table 1 shows the program of elderly public health care for the period of last 3 months that evaluated in this study.

### 2.4. Participation in the Elderly Health Program

The participation in the elderly health program was reported from secondary data, “subject visiting book”. Elderly community health care has operated for five years and held program activities 2-3 times per month continuity. This study recorded the number of visiting for 3 months; it means the maximum number of subject visits is eight times, and for statistical analysis, we divided based on the mean value.

### 2.5. Awareness

The awareness of salt intake was measured by asking the subjects using a simple self-description questionnaire “for the period of last 3 months, whether did you avoid or limit the consumption of salt?” The answer is only two options: they were yes or no. Yes answer means salt conscious, and no answer as nonsalt conscious.

### 2.6. Salt Preference

A simple questionnaire for salt preference included “Do you like salty foods?” and the subjects answered one from two options: they were yes or no. The fast and simple questionnaire was applied by Hashimoto et al. in 2008 [13].

### 2.7. Salt Intake

Since approximately 90% of sodium intake is excreted in the urine and salt excretion was considered roughly equal to sodium intake, we calculated and estimated salt intake based on twenty-four-hour urinary sodium excretion, and this was expressed as grams of sodium chloride per day [14]. The mean daily salt intake was calculated by the equation (Na (mmol/day) × 58.5)/1,000 = NaCl (g/day) [15]. For analysis, salt intake was divided based on Indonesian recommended dietary allowance (RDA) of salt intake for older women (3 g/day) and upper limit (6 g/day) [16] and also according to WHO recommendation 5 g/day [17]. Besides Indonesian RDA, the scientific advisory committee on nutrition (SACN) also gave recommendation of maximum salt intake for adults which is 6 g/day [18].

### 2.8. Blood Pressure

Blood pressure was measured once in the morning, the day of urine collection, using standardized technique and appropriately validated by the trained nurse. Measurements for three times using a standard calibrated mercury sphygmomanometer were obtained with participants, and the average of three readings was used for the analysis. We did not report BP from the activity program because of missing of accuracy. Blood pressure was measured by registered nurse trained using standard calibrated mercury sphygmomanometers in the morning, the day of urine collection. Hypertension was defined based on JNC 7 criteria as systolic BP (SBP) ≥140 mm Hg, diastolic BP (DBP) ≥90 mm Hg, a self-report of taking antihypertensive medication(s), or previously diagnosed by a physician.

### 2.9. Statistical Analysis

Kolmogorov–Smirnov test was used to check normality data. All statistical calculations were conducted with Statistical Package for Social Science (SPSS) version 21, and *p* value <0.05 was considered significant. Continuous variables were presented as mean and standard deviation, and categorical variables were summarized as numbers and percentages. Bivariate analysis to correlate continues variables was performed by Spearman test. On the contrary, chi-square test was performed to assess factors associated with salt intake, and logistic regression was used to determine the most factors influencing salt intake.

## 3. Results

Demographics of the 51 subjects are shown in Table 2. The most common subjects were living in the coastal area since birth, so the length of stay was almost similar to mean age 56.98 ± 5.7 years as 19 subjects (37.3%) were classified as hypertensive by measuring BP.

Salt intake was measured by urinary 24-hour, and the mean daily salt intake of all subjects was 6.16 ± 3.48 g/d.  Table 3 shows that approximately 62.8% of subjects had salt consumption <6 g/d because of the scientific advisory committee on nutrition (SACN) recommendation of maximum salt intake for adults of 6 g/d (17). However, only 11.8% of subjects consume salt <3 g/d. By the analysis of Spearman correlation, there was no significant correlation between sodium intake and BP (*p* > 0.05).

The evaluation of the public health program on salt intake in this study was done by getting information about awareness and participation, as well as the salt preference of the subjects.  Table 4 demonstrates the mean of visiting was 4.43 times from the total eight times of visiting. However, 74.5% of subjects showed preference to salty food as 68.6% of subjects obtained awareness that they must avoid salty food. Bivariate analysis showed subjects with the number of attendance less than 4 times have a risk to consume salt intake 4.75 times higher than subjects with high participation. Furthermore, subjects who have no awareness of salt intake at the moment have risk 6.36 higher to consume excessive salt than salt-conscious subjects. It can be seen that no variable is associated significantly with blood pressure.

## 4. Discussion

Studying about chronic disease especially hypertension (HT) is interesting and becomes public health priority since the prevalence of HT is still high enough especially in low- and middle-income countries including Indonesia [19]. Moreover, the prevalence of HT in our study (37.3%) is higher than Indonesian National Health Survey, and it is well known that dietary salt has been recognized to be major determinant of HT [4]. The gold standard method to assess salt intake is collecting 24-h urine collection although it is high cost and less feasible [12]. It is not easy to apply 24-h urine collection to measure daily salt intake in the healthy population particularly among elderly; our study faced difficulties for collecting 24-h urinary correctly; approximately a quarter of subjects must repeat to collect 24-h urinary until correct.

To our knowledge, this is the prior study evaluating daily salt intake using 24-h urine collection among the vulnerable population in Indonesia. Since the previous studies demonstrated that coastal area is known as risk factors of HT relating to intake of salty foods [11] and the elderly is the age group with the highest prevalence of HT [4], it is interesting to understand and analyze the food habit especially salt intake in the vulnerable population particularly the elderly community dwelling at coastal area.

The estimated dietary salt based on 24-h urinary salt excretion in this study was 6.16 ± 3.48 g/d; 60.78% of subjects were more than 5 g salt/day, the maximum amount recommended by WHO for adults [17, 20]. The salt intake in this study was over 74% of the adequate intake level by Dietary Reference Intake for Indonesia [21]. Epidemiological study showed the vast majority of the world's citizens, everywhere, consume salt intake higher than general recommendation that limits salt intake to 6 g/day [20]. It indicated that salt intake in our study is still categorized high; only 11.8% of subjects consumed salt intake <3 g/day. The American Heart Association (AHA) advocates for declining in sodium consumption in the U.S. diet to 1500 mg/day by 2020; it is nearly the same as 3-4 g salt dietary [7]. Reductions in dietary salt to 3 g of salt per day could significantly reduce cardiovascular events and medical cost and yield substantial reductions in morbidity, mortality, and health care costs [6]. Urban coastal dweller is categorized as the vulnerable group since a geographical location such as coastal area is known as risk factors of hypertension relating to the high exposure of salty food [9]. Recent study demonstrated that housewives and those living in the coastal area had a significantly higher probability of high daily salt intake compared with people living in hilly areas [22]. However, it should be noted that majority of subjects (62.8%) consume salt < 6 g/d; it means 62.8% subjects can maintain salt intake allowing dietary recommendation. As can be seen, these findings were lower than studies in Asian (average mean salt intake >12 g/day) and Western countries with the mean salt intake of >6 g/day [20]. Same trend was observed in The International Study of Salt and Blood Pressure (INTERSALT), which included more than 10,000 subjects from 32 countries and showed urinary salt excretion of around 9.9 g of salt per day [23].

Public health access may have an important role for maintaining salt intake in this study. Public health care, the leading health sector in the community, has an important role to promote population-level salt reduction by raising consumer awareness regarding salt intake [9, 10]. It is strongly supported by the previous study that creating awareness about the implication of excessive salt intake on health and developing strategies for reducing salt intake was reasonably possible to be implemented at the community level [22]. Our analysis revealed 68.6% of subjects avoided salty food though actually 74.5% subjects like to salty taste. Moreover, awareness of salt intake was associated with actual salt intake (*p*=0.005). Our finding clearly demonstrated subjects had awareness that salty food must be avoided. This is confirmed by the previous study that customer awareness and education interventions resulted in a reduction in 24-hour urinary sodium excretion [10].

Coastal area in our study was located in the urban central city so the accessibility of health information and health care services could be achieved easily. Availability of health care providers clearly influences access to health care, treatment, and rehabilitation for chronic conditions. One of the important external barriers which limits access to health services is the actual lack of available services, so it is crucial to improve the accessibility of health service as well as those services being available and easily accessed [24]. Located in the metropolitan city and near from the center of health service and education centers, the urban coastal area in our study is very available to be visited by health care providers. The information about the health problem and its risk factors including to limit salt intake can be easy achieved. Our study demonstrated that the elderly care program has developed for 5 years and held activities regularly 2-3 times a week by giving health education, training/exercise for the elderly, and supplementary feeding. In addition, nutrition education about salt intake or salty food is the most common material given by health care providers. Previous studies demonstrated that awareness of salt restriction may be due to the professional nutritional guidance [25].

The public health access includes geographical distance, the availability of health care providers, and rural culture [9]. The average length of residence of respondents was similar to mean age (52.8 ± 12.57) because almost all respondents lived in coastal areas from birth. They lived in surrounding or near from community health centers; the longest distance is only about 2 kilometers, and majority of subjects only walked to get that place. It means that our subjects can easily access to achieve the health care service. The recent study in rural coastal in Indonesia demonstrated the prevalence of HT by 40.4%. The finding is higher than that in our study; it is plausible that accessibility to health care is so far [26]. The meaningful reason is also shown by a previous study which also demonstrated that traveling long distances to health care facility can cause hard to reach, particularly if transportation is difficult or the weather is bad [9].

The easy access to get health care might subject to participate in the program and has an important role to public health outcomes [8, 9]. Our study revealed that participation was associated significantly with salt intake; low participation has risk 4.75 higher to consume excessive salt than that of high participation. Furthermore, there was significant association between awareness and participation with *p*=0.023 (data were not shown). To support this finding, a recent study in Indonesia found the salt restriction and efficacy-maintenance program was effective in improving knowledge, attitudes, and self-efficacy of salt-reduction practices and could be applied with community-dwelling older people with high blood pressure [27]. Effectively reducing the salt intake among hypertensive female elderly by improving nutrition knowledge, dietary behavior, and finally adherence to a recommendable low-sodium diet was also shown by a previous study [28].

Several countries have already successfully reduced salt intake in their populations including Japan, Finland, and more recently the United Kingdom [7]. Hence, it is not impossible to reduce salt intake in the vulnerable population. The easy access and low salt prices, as well as a conductive environment might cause respondents to limit their salt consumption. However, the fact is the prevalence of HT in our study was still high; furthermore, there was no association between the public health program including awareness, participation, and hypertension. This finding differs from the previous study that the access to healthcare is associated with health outcomes [8]. Meta-analysis study showed how public health access had significant effect on awareness and health improvement including hypertension [9]. The improved access will increase the request for care and support the optimal service for chronic disease treatment [29]. Another study showed easy access to medical care is the key strategy which has proved to be successful in reducing hypertension at the population level [30]. However, our finding is similar to Rubinstein et al.'s study that health intervention in prehypertension subjects did not result in a change in BP but only was associated with an improvement in some dietary habits [31]. Moreover, distance traveled to clinic visit was also not associated with the therapeutic success of controlling the blood pressure [32].

Many factors are associated with blood pressure involving the complex mechanism especially in the older people. Systematic review studies showed that a higher proportion of uncontrolled blood pressure was associated with nonadherence to medication [33–35]. The study in Indonesian primary health care reported high prevalence of polypharmacy among the geriatric population, and it was associated with nonadherence [36]. Hormonal decline is the most important thing among the elderly, and it is a characteristic of the menopause along with an elevated blood pressure by activation of the sympathetic nervous system and the renin-angiotensin system [37]. Since daily salt intake is still significantly high and hypertension is still prevalent, comprehensive strategies to reduce salt such as reduction of sodium content in the packaged food, environmental recondition, and product reformulation should be considered in development of sodium-reduction initiatives in this region [17].

Potential limitation should be noted since we do not know exactly the impact of the public health program on reducing salt intake in our subjects. We did not measure salt intake and blood pressure of subjects before the PH program started, and we did not control several confounding variables influencing outcome (hypertension) such as other diseases suffered by the elderly. Furthermore, the design of our study is cross sectional and thus cannot address causality or directionality of association. However, the strength of this study is we applied a gold standard to identify and measure salt intake in the population using 24-h urinary excretion.

## 5. Conclusions

Our study highlights salt intake among older women in the coastal area was still categorized high; only 11.8% of subjects consumed salt intake <3 g/day. However, majority of subjects (62.8%) consume salt < 6 g/d. There was a significant association between participation, awareness, and salt intake which might suggest that participating regularly in the health care program and increasing awareness on the harmful effects of excessive salt consumption may cause our subjects limit their salt consumption. Since daily salt intake is still significantly high and hypertension is still prevalent, comprehensive strategies to reduce salt intake should be considered in development of sodium-reduction initiatives in this region.

## Figures and Tables

**Table 1:** Evaluation of salt intake at elderly public health program.

Variable
Program evaluation
(i) Program was held 2-3 times/month. We evaluated for 8 times for 3 months.
(ii) Program involved interprofessional health physicians such as nurses, general practitioners, nutritionists, and health counselors

The activities of the program
(i) Regular physical examination
(ii) Measurement of blood pressure
(iii) Drug administration including antihypertension drugs
(iv) Health or nutrition education
(v) Physical activities/light training
(vi) Supplementary feeding

Subject evaluation
(i) Number of attendance (participation) from the subject visiting book
(ii) Awareness of salt intake by asking some simple questions
1. At the present time, do you avoid salt intake?
2. Do you think that salt intake is harmful for the healthy particularly hypertension?
3. Do you get information about salt intake from the health education program?

**Table 2:** Demographic variables of the 51 subjects who participated in the study.

Variables	Mean ± SD or *n* (%)
Continuous variables	
Age (year)	56.98 ± 5.7
Length of stay (year)	52.8 ± 12.57
Body mass index (kg/m^2^)	25.96 ± 4.85
Systolic blood pressure (mm Hg)	132.25 ± 17.78
Diastolic blood pressure (mm Hg)	83.63 ± 10.3

Categorical variables	
Blood pressure	
Norm tension	32 (62.7%)
Hypertension	19 (37.3%)
Education level	
Elementary school	34 (66.7%)
Junior high school	12 (23.5%)
Senior high school	5 (9.8%)
Consume antihypertensive drug	
Yes	15 (29.4%)
No	36 (70.59%)

**Table 3:** Estimated salt intake from 24-h urinary stratified by Indonesian RDA and WHO and the correlation with blood pressure.

Variables	
Mean ± SD (g/day)	6.16 ± 3.48

Based on Indonesian RDA	
<3 g/d	6 (11.8%)
3–6 g/d	26 (51%)
>6 g/d	19 (37.2%)

Based on WHO recommendation	
≤5 g/d	21 (41.2%)
>5 g/d	30 (58.8%)

Adequacy salt intake (3 g/d)	205.38 ± 116.17%

Adequacy salt intake (5 g/d)	123.23 ± 69.7%

Adequacy salt intake (6 g/d)	102.69 ± 58.09%

Bivariate analysis	
Salt intake and systolic BP	*p*=0.71
Salt intake and diastolic BP	*p*=0.41

^*∗*^Spearman correlation and *p* < 0.05 is considered significant.

**Table 4:** Bivariate analysis for factors associated with salt intake and hypertension status.

Variable	Salt intake					Blood Pressure	
	Total (51) *n* (%)	Low (<6 g)	High (≥6 g)	P^1^	Normotension	Hypertension	P^1^
Number of attendance (participate)	4.43 ± 1.37						
>Mean (5–8)	24 (47.06)	19	5	0.011^*∗*^	17	7	0.26
<Mean (1–4)	27 (52.94)	12	15	OR 4.75 (1.37–16.48)	15	12	

Awareness (avoid salty food)							
Yes	35 (68.63)	26	9	0.003^2^^*∗*^	24	11	0.2
No	16 (31.37)	5	11	OR 6.36 (1.73–23.34)	8	8	

Preference to salty food							
Yes	38 (74.51)	22	16	0.47	24	14	0.91
No	13 (25.49)	9	4		8	5	

^1^Chi-square test; ^*∗*^*p* < 0.05 is considered significant.

## Data Availability

The data used to support the findings of this study are available from the corresponding author upon request and are restricted by the ethics of Faculty of Public Health, Universitas Airlangga, in order to protect subject privacy.
